# The Epidemiology of Spondylodiscitis in Germany: A Descriptive Report of Incidence Rates, Pathogens, In-Hospital Mortality, and Hospital Stays between 2010 and 2020

**DOI:** 10.3390/jcm12103373

**Published:** 2023-05-09

**Authors:** Siegmund Lang, Nike Walter, Melanie Schindler, Susanne Baertl, Dominik Szymski, Markus Loibl, Volker Alt, Markus Rupp

**Affiliations:** 1 Department for Trauma Surgery, University Medical Center Regensburg, Franz-Josef-Strauss-Allee 11, 93053 Regensburg, Germany; 2 Centrum für Muskuloskeletale Chirurgie, Universitätsmedizin Berlin, Charitéplatz1, 10117 Berlin, Germany; 3 Department of Spine Surgery, Schulthess Clinic Zurich, Lenghalde 2, 8008 Zurich, Switzerland

**Keywords:** spondylodiscitis, mortality, pathogens, antimicrobial resistance, epidemiology, geriatric population

## Abstract

Background: Spondylodiscitis can lead to significant morbidity and mortality. Understanding its up-to-date epidemiological characteristics and trends is important to improve patient care. Methods: This study analyzed trends in the incidence rate of spondylodiscitis cases in Germany between 2010 and 2020, as well as the pathogens, in-hospital mortality rate, and length of hospital stay. Data were obtained from the Federal Statistical Office and the Institute for the Hospital Remuneration System database. The ICD-10 codes “M46.2-”, “M46.3-” and “M46.4-” were evaluated. Results: The incidence rate of spondylodiscitis increased to 14.4/100,000 inhabitants, with 59.6% cases occurring in patients 70 years or older and affecting mainly the lumbar spine (56.2%). Absolute case numbers increased from 6886 by 41.6% to 9753 in 2020 (IIR = 1.39, 95% CI 0.62–3.08). Staphylococci and *Escherichia coli* were the most coded pathogens. The proportion of resistant pathogens was 12.9%. In-hospital mortality rates increased to a maximum of 64.7/1000 patients in 2020, intensive care unit treatment was documented in 2697 (27.7%) cases, and the length of stay per case was 22.3 days. Conclusion: The sharply increasing incidence and in-hospital mortality rate of spondylodiscitis highlights the need for patient-centered therapy to improve patient outcomes, especially in the geriatric, frail population, which is prone to infectious diseases.

## 1. Introduction

Musculoskeletal infections represent a major challenge in orthopedic and trauma surgery [1]. Infections of the spine caused by pyogenic bacteria without prior surgery or implant insertion involving the intervertebral disc and vertebral bodies are referred to as (pyogenic) spondylodiscitis [2]. Patients suffering from spondylodiscitis commonly need to be hospitalized [3,4,5,6]. Insidious courses, especially in infections with low virulent pathogens, often lead to delayed diagnosis, which can be associated with high morbidity and mortality [7]. In this context, the significance of infections with coagulase-negative staphylococci (CONS) must be increasingly emphasized [8,9,10]. Even after completing treatment, patients tend to suffer relevant impairment in quality of life [11]. Epidemiological studies have observed rising incidence rates of spondylodiscitis in Europe, indicating an ongoing challenge for stakeholders in healthcare systems [12,13,14]. In general, the treatment of elderly patients, who are especially prone to infections, will keep gaining importance for musculoskeletal surgeons [15]. On the one hand, increasing numbers of comorbidities of an aging population can be assumed as potential reasons for this development [16,17]. Conversely, advancements in imaging technology have led to improved diagnostic capabilities, resulting in an increased number of documented cases of spondylodiscitis [18,19]. Lastly, the standardization in pathogen identification methods has emerged and most likely further contributed to the detection of spondylodiscitis [20,21]. Up-to-date diagnostic and therapeutic measurements in spondylodiscitis treatment recently have been summarized [22,23]. Nevertheless, considerable heterogeneity in reports on the epidemiology of spondylodiscitis persists, analyses of nationwide databases are sparse and the rate of in-hospital mortality remains to be elucidated [12,14,24,25].

Therefore, the aim of this study was (1) to determine the epidemiological characteristics of pyogenic spondylodiscitis and the development of the nationwide incidence in adults from 2010 through 2020. (2) The second aim was to provide a comprehensive overview of the pathogens documented as concomitant diagnoses in 2020. (3) Lastly, we aimed to analyze the development of the in-hospital mortality rate, duration of hospitalization and proportion of cases which required intensive care unit (ICU) treatment between 2010 and 2020.

## 2. Materials and Methods

### 2.1. Federal Statistical Office of Germany (Destatis)

Data consisting of annual codes of the tenth version of the International Statistical Classification of Diseases and Related Health Problems (ICD-10) diagnosis codes from German medical institutions between 2010 and 2020 were provided by the Federal Statistical Office of Germany (Destatis). The total number of spondylodiscitis cases was quantified by adding ICD-10 codes “M46.2-”, “M46.3-” and “M46.4-” and analyzed as a function of sex and age in 10-year increments for patients older than 20 years between 2010 and 2020. Incidence rates were calculated based on Germany’s historical population aged 20 years or older provided by Destatis. Here, the number of inhabitants in each of the 16 German federal states was considered by year of birth for each year of the period 2010 through 2020. The deadline of each year was December 31. Incidence rate ratios (IIR) with the corresponding 95% confidence interval (CI) and percentage changes were calculated by dividing the incidence in 2020 by the incidence of the preceding year for all spondylodiscitis cases. Changes in the incidence rate ratios were determined relative to the year 2010. Furthermore, Destatis provided the number of in-hospital deaths and number of hospitalization days for spondylodiscitis cases during the observation period.

### 2.2. Institute for the Hospital Remuneration System (InEK GmbH)

In accordance with Section 17b of the German Hospital Financing Act, a universal, performance-based, and flat-rate remuneration system has been introduced for general hospital services. The basis for this is the German Diagnosis Related Groups system (G-DRG system), whereby each inpatient case of treatment is remunerated through a corresponding DRG lump sum payment. The InEK GmbH provides detailed data on the main diagnoses (based on ICD-10 codes), age and sex distribution, length of hospital stays, reasons for discharge (including “death”), number of intensive care unit (ICU) cases and coded concomitant diagnoses (based on ICD-10 codes) (22). The InEK browser enables analysis back to the year 2019. The following comprehensive analysis was made only for the year 2020. Based on the ICD-10 codes for spondylodiscitis, as listed above, data for total case numbers, numbers of pathogens coded as concomitant diagnoses, number of in-hospital deaths, and the number of cases with ICU treatment were extracted. Informed Consent and Investigational Review Board (IRB) approval was not required for this study as it used data from anonymous, de-identified, administrative databases.

## 3. Results

### 3.1. The Development of the Nationwide Incidence of Spondylodiscitis from 2010 to 2020

**Figure 1:**
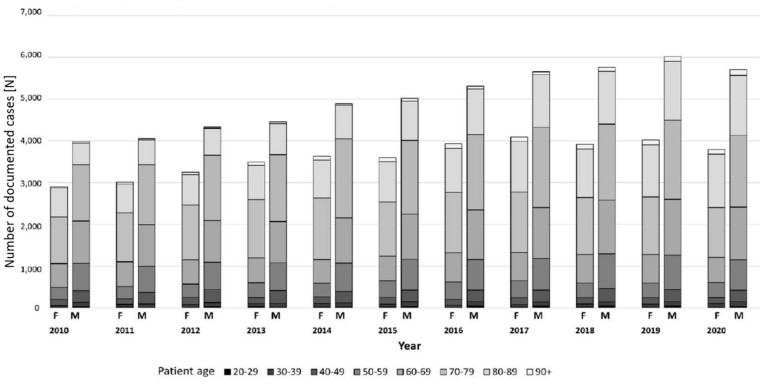
Age and gender distribution of Spondylodiscitis cases between 2010 through 2020 in absolute numbers.

**Figure 2:**
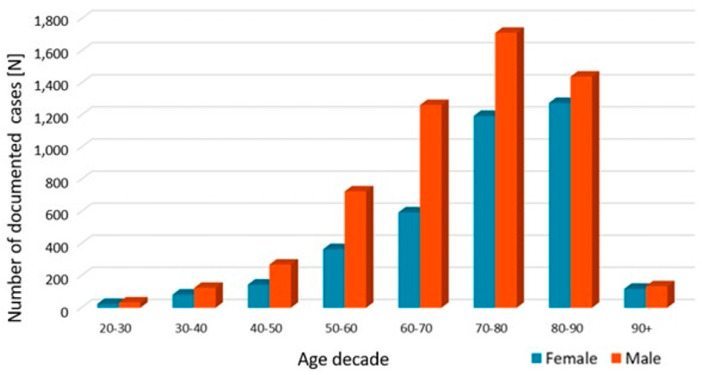
Age and gender distribution of spondylodiscitis cases in 2020 in absolute numbers.

**Figure 3:**
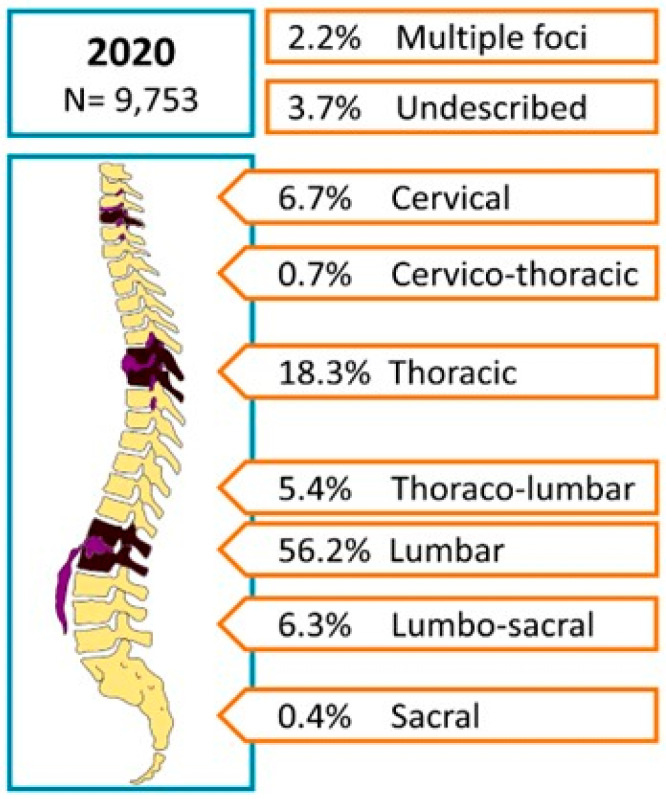
The distribution of spondylodiscitis by anatomical location of 9753 InEK cases in 2020 (designed with Inkscape and Microsoft PowerPoint).

In total 95,075 in-hospital spondylodiscitis cases were registered between 2010 and 2020. In 2010, a total number of 6886 cases were listed in Germany, constituting an annual incidence of 10.4 cases per 100,000 inhabitants (95% CI 10.1–10.6). In the following years, the incidence rose steadily, resulting in a maximum of 14.8 cases per 100,000 inhabitants (95% CI 14.5–15.1) in 2019. In 2020, the number slightly decreased to an incidence of 14.4 per 100,000 inhabitants (95% CI 14.1–14.7). Compared to the year 2010, absolute case numbers increased by 41.6% (IIR 1.39, 95% CI 0.62–3.08). Of all the cases analyzed, the majority (59.6%) occurred in patients aged 70 years or older. Male patients constituted 58.2% of the cohort. In 2020, the male/female ratio was 1.5. No relevant changes in the male/female ratios were observed in the 11 years period (Table 1, Figure 1). The proportion of patients 70 years or older increased from 54.4% in 2010 to 62.2% in 2020 (Table 1, Figure 2). In 2020, most frequently, spondylodiscitis was present in the lumbar spine (56.2%), followed by the thoracic spine (18.3%), and the cervical spine (6.7%). Spondylodiscitis with multiple foci was documented in 2.2% of the cases (Figure 3).

### 3.2. Pathogens Codes in Spondylodiscitis Cases in 2020

Pathogens were coded for as concomitant diagnosis in 7589 cases (77.8%) in 2020. It could not be differentiated if these pathogens were exclusively causative for spondylodiscitis. In most cases “other/unspecified staphylococci” were documented (27.1%), followed by *E. coli* and other Enterobacterales (22.4%), *Staphylococcus aureus* (*S. aureus*) (19.4%) and streptococci (17.8%). *Pseudomonas* spp. and other non-fermenters were documented in 5.2% of the cases (Table 2). In 12.9% of cases where pathogens were identified, codes indicating resistance to antimicrobial drugs were applied. The most commonly identified resistant pathogen was *Enterococcus faecium* with resistance against glycopeptide antibiotics (U80.30; 2.8% of all cases), followed by methicillin-resistant *S. aureus* (U80.00; MRSA; 1.9%), and 3MRGN *E. coli* (U81.20; 1.6%). Overall, Gram-positive pathogens made up 65.0% of all resistant pathogens.

### 3.3. In-Hospital Mortality Rate, Hospitalization and ICU Treatment

The in-hospital mortality rate in spondylodiscitis cases increased from 45.6 per 1000 patients (95% CI 40.6–50.6) to a maximum of 64.7 per 1000 patients (95% CI 59.6–69.7) in 2020. The absolute number of in-hospital deaths rose by 101.0% from *n* = 314 in 2010 to *n* = 631 in 2020 (Table 3). The total number of inpatient treatment days in 2010 was 173,581, corresponding to a calculated hospital length of stay of 25.2 days per case. By 2020, the total number of hospital days increased to 217,416 (+25.3%). Given the increasing number of spondylodiscitis cases, the calculated hospital length of stay per case was 22.3 days in 2020. The maximum number of hospital days was recorded in 2019 at 230,093 days (22.9 days per case). In 2020, ICU treatment was documented in 2697 (27.7%) spondylodiscitis cases. The mortality rate of ICU cases was 165.0 per 1000 patients. Among spondylodiscitis patients who received treatment in an ICU, 72.5% were aged 65 years or older. The percentage ratio of male to female patients was 63/37. The mean hospitalization time of ICU cases was calculated to be 31.3 ± 26.6 days in 2020.

## 4. Discussion

As a main finding of this cross-sectional study, we report a significant increase of spondylodiscitis cases in the adult German population by 41.6% between 2010 and 2020. While studies relying on data from single hospitals may yield skewed results, the findings presented here are based on nationwide reports from the largest country of the European Union. The study provides a comprehensive, historical overview of the age and gender distribution, in-hospital mortality rate, and hospitalization time of spondylodiscitis cases. It further presents the coded pathogens, anatomical site of infection, and characteristics of cases treated in an ICU in 2020.

### 4.1. The Development of the Nationwide Incidence of Spondylodiscitis between 2010 and 2020

Increasing rates of spondylodiscitis cases have been reported by several authors during the last decades [12,13,14,26]. The majority of reports on pyogenic spondylodiscitis introduce an estimated incidence of 0.2 to 2.4/100,000 per annum, mostly based on the analysis of Grammatico et al. from 2002 to 2003 [13]. Most recently, Conan et al. reported an increase in spondylodiscitis incidence from 6.1/100,000 in 2010 to 11.3/100,000 (+46%) in 2019 based on a French nationwide database, including 42,105 hospitalized patients [14]. Similarly, the current study demonstrated an increase in the spondylodiscitis incidence in the adult German population of 41.6% during the 11-year study period including 95,075 spondylodiscitis cases; the incidence was 14.4 per 100,000 inhabitants in 2020. Consistent with the literature, the male-to-female ratio was 1.5 in 2020, and the analysis demonstrated no relevant change in the sex distribution during the 11-year observation period [13,14]. It must be assumed that an improvement and better availability of CT and MRI diagnostics may have contributed to a higher detection rate of spinal infections, comparable to the trend in vertebral insufficiency fractures [27]. The enhanced awareness and recognition of spondylodiscitis among healthcare professionals could also lead to more accurate and timely diagnoses, resulting in a higher reported incidence [22]. However, it can be assumed that an important reason for the rising incidence is the aging population, which suffers from an increasing number of comorbidities [9,16]. The increasing prevalence of risk factors, such as diabetes mellitus, obesity, and immunosuppression, which predispose individuals to infections including spondylodiscitis has been underlined by Kremers et al., who demonstrated a significant increase in osteomyelitis cases in their population-based study on 760 incident cases [28]. Mavrogenis et al. presumed that a rise in the susceptible population and improved diagnosis capabilities have increased the reported incidence of the disease in recent years [29]. This thesis is underlined by the reported age distribution in the current study; of all cases, 59.6% were 70 years or older. We demonstrated that the proportion of patients aged 70 years or older increased from 54.4% in 2010 to 62.2%. Furthermore, the growing number of invasive spinal procedures and interventions, such as spinal instrumentation surgeries, may be associated with a higher risk of postoperative or interventional spondylodiscitis, and consequently contribute to the observed increase in overall cases [30,31]. Interestingly, the incidence rate of spondylodiscitis decreased from 2019 to 2020 by 2.7%, against the identified rising trend in the previous years. Bearing in mind the decimating effect on the hospitalization rates in orthopedic and trauma surgery, it can be hypothesized that the COVID-19 pandemic may have contributed to this development [32,33].

### 4.2. Pathogens

Contradictory to the body of literature, we found staphylococci other than *S. aureus* to be identified most frequently, followed by *E. coli* and Enterobacterales. The high prevalence of other staphylococci suggested a high share of CONS. This is in line with recent reports on the growing importance of the role of healthcare-associated infections, in particular device-associated bloodstream infections for the etiology of spondylodiscitis [9,34]. Kim et al. demonstrated in a retrospective review of 586 culture-confirmed spondylodiscitis case an age-dependent prevalence of causative pathogens. *S. aureus* was more common in patients aged <60 years (53.7%), whereas Gram-negative bacteria were more common in patients aged 60 years or older (30.9%, vs. 14.7% in patients aged <60 years) [35]. They further showed an association between Gram-negative pathogens in patients with liver cirrhosis and solid tumors [35]. Interestingly, *S. aureus* was only documented in 19.4% of cases with identified pathogens. This contradicts the vast majority of literature, reporting *S. aureus* as the most common causative pathogen in around 50% of spondylodiscitis cases [24,36]. Notable, Conan et al. reported comparable pathogen prevalence in their up-to-date, nationwide study; all staphylococci accounted for 55.0% of pathogens (46.5% in our results), Gram-negative pathogens for 21.1% (22.4%) and streptococci for 10.2% (17.8%) [14]. This may suggest increasing importance of Gram-negative pathogens and CONS, mirroring the epidemiological development towards older, frail patients. Reports on (multi-resistant) pathogens in spinal and musculoskeletal disease vary widely, and they depend on the socio-economic and geographical background of the cohort. In the current study, codes for pathogens with resistance against antimicrobial drugs were applied in 12.9% of cases with identified pathogens. [37,38,39]. The most common resistant pathogen was *Enterococcus faecium* with resistance against glycopeptide antibiotics, second was methicillin-resistant *S. aureus* and third 3MRGN *E. coli*. This distribution is concordant with the recent development of pathogen resistance in Germany, where *Enterococcus faecium* shows rising resistance rates against Vancomycin [40]. In contrast, the rates of 3MRGN *E. coli* and MRSA are decreasing [40]. The rise in antibiotic-resistant pathogens could contribute in the complexity of spondylodiscitis treatment, potentially leading to increased morbidity and mortality rates [26,41].

It must be pointed out that the coding quality for pathogens is usually low. Furthermore, it could not be differentiated if the pathogens documented as a concomitant diagnosis in the InEK database were causative for spondylodiscitis in the current analysis, which therefore must be interpreted carefully.

### 4.3. In-Hospital Mortality Rate, Hospitalization Time and ICU Treatment

Following the Europe-wide trend [42], the average length of stay of spondylodiscitis patients decreased from 25.2 days in 2010 to 22.3 days in 2020. Thisstill f exceeds the mean length of stay of 8.7 days of all hospitalized patients in Germany in 2020 by far [42]. We demonstrated an increase in the in-hospital mortality rate from 45.6 per 1000 patients to a maximum of 64.7 per 1000 patients in 2020, a 101.0% increase in the total number. The high proportion of 27.7% of cases treated in the ICU, with a mortality rate of 165.0 per 1000 patients, highlights the severity of the disease. In a recent retrospective cohort study on 155 patients with pyogenic vertebral osteomyelitis, we found an in-hospital mortality rate of 12.9% [8]. Even higher mortality rates are reported in the mid-to-long-term follow-up in the literature; Yagdiran et al. reported a 1- and 2-year mortality rate of 20% and 23%, respectively [6]. Vettivel et al. reported a mortality rate of 5.2% at 30 days and 22.3% at one year in a single-center study on 76 patients with pyogenic vertebral osteomyelitis. The high incidence of the concomitant disease is likely a major factor in the risk of mortality in the elderly population that is susceptible to developing spondylodiscitis. Kehrer et al. showed a 1-year mortality rate of 20% in a cohort of 298 patients [43]. They claimed that the main factors associated with short-term mortality were severe neurologic deficits at the time of admission, epidural abscess and comorbidities [43]. In a nationwide Danish study Aagaard et al. found that spondylodiscitis patients have increased long-term mortality, mainly due to comorbidities, particularly substance abuse [17]. The current study did not consider concomitant diagnoses or treatment aspects as factors influencing mortality rates. In addition, the data sources do not allow for an evaluation outside the inpatient sector. Potential risk and protective factors for spondylodiscitis mortality remain to be investigated nationwide.

### 4.4. Strengths and Limitations

An outstanding characteristic of this epidemiological evaluation is that the analysis is based on registry data consisting of ICD-10 diagnosis codes from all German medical institutions showing the development of the incidence rate of spondylodiscitis over eleven years. The main limitation of this study is that it represents a purely descriptive report and thus, a lack of statistical analysis beyond incidence rate ratios and corresponding 95% confidence intervals must be acknowledged. Only inpatient data were available. In addition, correct coding cannot be assured. A detailed annual analysis was conducted only for the primary diagnoses because the InEK data browser only enables analysis of concomitant diagnoses back to 2019. Furthermore, this analysis does not provide information on the treatment modalities such as surgery or antimicrobial therapy. Our data source does not provide these details, but their relevance in understanding spondylodiscitis management must be emphasized. Future studies should consider these aspects to provide a more comprehensive view of the condition and its treatment.

## 5. Conclusions

Between 2010 and 2020 the incidence rate of hospitalized spondylodiscitis cases increased notably to 14.4 per 100,000 adult inhabitants in Germany. This trend primarily affected elderly men. It is important to consider that factors such as an aging population, more frequent medical interventions and improved diagnostic techniques could have contributed to the rising incidence. Despite advances in treatment strategies, the average in-hospital mortality increased during the observation period and the rate of ICU treatments was high. The epidemiological trend observed in this study is consistent with nationwide developments. It is crucial to emphasize the need for patient-centered therapy, specifically tailored to the elderly and frail population, while taking into account the evolving spectrum of pathogens. Further research is needed to understand the underlying causes of this increase in incidence and to optimize treatment and preventive strategies for spondylodiscitis.

## Figures and Tables

**Table 1:** Historic development of spondylodiscitis diagnoses between 2010 and 2020.

Year	Total Number	German Population 20 Years or Older	Change in Total Numbers (Relative to 2010)	Incidence per 100,000 Inhabitants	Incidence Relative to the Preceding Year	Incidence Rate Ratio Relative to the Preceding Year [95% CI]	Ratio of Female/Male	Ratio Aged ≤70 Years/>70 Years
2010	6886	66,549,975	-	10.4 [10.1–10.6]	-	-	42/58	46/54
2011	7067	65,398,514	2.6%	10.6 [10.4–10.9]	2%	1.02 [0.44–2.41]	43/57	44/56
2012	7582	65,665,069	10.1%	11.4 [11.3–11.9]	7%	1.07 [0.47–2.48]	43/57	43/57
2013	7946	65,943,867	15.4%	12.2 [11.8–12.4]	7%	1.07 [0.48–2.39]	44/56	41/59
2014	8519	66,677,665	23.7%	13.0 [12.6–13.2]	7%	1.07 [0.49–2.34]	43/57	39/61
2015	8618	67,097,676	25.2%	13.1 [12.7–13.2]	1%	1.01 [0.47–2.17]	42/58	40/60
2016	9243	67,440,230	34.2%	13.9 [13.4–14.0]	6%	1.06 [0.50–2.26]	43/57	40/60
2017	9749	67,540,025	41.6%	14.5 [14.1–14.7]	4%	1.04 [0.50–2.18]	42/58	38/62
2018	9677	67,724,921	40.5%	14.3 [14.0–14.6]	−1%	0.99 [0.48–2.06]	40/60	40/60
2019	10,035	67,864,036	45.7%	14.8 [14.5–15.1]	3%	1.03 [0.50–2.14]	40/60	39/61
2020	9753	67,820,457	41.6%	14.4 [14.1–14.7]	−3%	0.97 [0.62–3.08]	40/60	38/62

**Table 2:** Pathogens documented as concomitant diagnosis of spondylodiscitis cases in 2020 (sorted by frequency).

ICD-10 Code	Pathogen (Coded as Concomitant Diagnosis)	Number of Cases	Percentage of Cases with Pathogen	Cumulative Percentage	Stratified Pathogens
B95.7	Other staphylococci.	1258	16.6%	27.1%	Other/unspecified staphylococci
A49.0	Staphylococcal infection of unspecified location.	722	9.5%		
B95.8	Unspecified staphylococci.	78	1.0%		
B96.2	*Escherichia coli* (*E. coli*) and	1702	22.4%	22.4%	*E. coli* and Enterobacterales
	other Enterobacterales.				
B95.6	*S.s aureus*.	1472	19.4%	19.4%	*S. aureus*
B95.2	Streptococci, group D,	840	11.1%	17.8%	Streptococci
	and enterococci.				
B95.48	Other specified streptococci.	310	4.1%		
B95.1	Streptococcus, group B.	94	1.2%		
B95.5	Unspecified streptococci.	46	0.6%		
B95.3	Streptococcus pneumoniae.	19	0.3%		
B95.41	Streptococcus, group C.	17	0.2%		
B95.42	Streptococci, group G.	16	0.2%		
B95.0	Streptococcus, group A.	11	0.1%		
B96.5	*Pseudomonas* and other nonfermenters.	395	5.2%	5.2%	Pseudomonas and other nonfermenters
B95.91	Other specified Gram-positive, anaerobic, non-spore-forming pathogens.	243	3.2%	4.8%	Other Gram-positive pathogens
B95.90	Other specified Gram-positive	124	1.6%		
	aerobic pathogens.				
B96.8	Other specified bacteria.	114	1.5%	3.2%	Other
B96.6	*Bacteroides fragilis* and other Gram-negative anaerobes.	64	0.8%		
B96.7	*Clostridium perfringens* and other Gram-positive, spore-forming anaerobes.	26	0.3%		
B98.0	*Helicobacter pylori* (*H. pylori*).	20	0.3%		
B96.3	Haemophilus and Moraxella.	18	0.2%		

**Table 3:** Historic development of in-hospital deaths of spondylodiscitis cases between 2010 and 2020.

Year	Total Number	German Population Aged 20 Years or Older	Change in Total Numbers (Relative to 2010)	In-Hospital Mortality Rate per 1.000 Patients [95% CI]	Incidence Rate Ratio Relative to the Preceding Year [95% CI]
2010	314	6886	-	45.6 [40.6–50.6]	1.0 [0.6–1.4]
2011	307	7067	−2.2%	43.4 [38.6–48.3]	1.0 [0.6–1.5]
2012	314	7582	0.0%	41.4 [36.8–46.0]	1.2 [0.8–1.8]
2013	393	7946	25.2%	49.5 [44.6–54.3]	1.0 [0.7–1.4]
2014	409	8519	30.3%	48.0 [43.4–52.7]	1.2 [0.8–1.7]
2015	478	8618	52.2%	55.5 [50.5–60.4]	1.0 [0.7–1.5]
2016	513	9243	63.4%	55.5 [50.7–60.3]	1.1 [0.8–1.6]
2017	596	9749	89.8%	61.1 [56.2–66.0]	1.0 [0.7–1.4]
2018	577	9677	83.8%	59.6 [54.8–64.5]	1.0 [0.7–1.4]
2019	585	10,035	86.3%	58.3 [53.6–63.0]	1.1 [0.8–1.6]
2020	631	9753	101.0%	64.7 [59.6–69.7]	1.4 [1.0–2.1]

## Data Availability

All data presented in this study are available on demand from the corresponding author.
